# An Evaluation of the Collagen Fragments Related to Fibrogenesis and Fibrolysis in Nonalcoholic Steatohepatitis

**DOI:** 10.1038/s41598-018-30457-y

**Published:** 2018-08-17

**Authors:** Yi Luo, Abdul Oseini, Robert Gagnon, Edgar D. Charles, Kurex Sidik, Robert Vincent, Rebeca Collen, Michael Idowu, Melissa J. Contos, Faridoddin Mirshahi, Kalyani Daita, Amon Asgharpour, Mohammed S. Siddiqui, Gabor Jarai, Glenn Rosen, Rose Christian, Arun J. Sanyal

**Affiliations:** 1 Fibrosis Translational Research and Development, Bristol-Myers Squibb, Pennington, NJ USA; 2 Division of Gastroenterology, Hepatology and Nutrition, Virginia Common Wealth University, Richmond, VA USA

## Abstract

Fibrosis, resulted from the imbalance of fibrogenesis and fibrolysis, is a key readout of disease progression in nonalcoholic steatohepatitis (NASH) and reflects mortality risk. Non-invasive biomarkers capable of diagnosing fibrosis stages and monitoring fibrosis changes in NASH patients are urgently needed. This study is to evaluate collagen formation and degradation biomarkers, reflective of fibrogenesis or fibrolysis, in patients with biopsy proven NASH. Collagen formation biomarker PRO-C3 and PRO-C6 levels were significantly higher in patients with advanced fibrosis stage 3–4 than those with fibrosis stage 0–2. Elevated PRO-C3 levels were also associated with severe lobular inflammation and ballooning, but not with steatosis. Multivariate logistic regression analysis identified PRO-C3 and PRO-C6 to be independently related to fibrosis stage. PRO-C3 showed similar performance to identify patients with advanced fibrosis in discovery and validation cohorts. Furthermore, in a longitudinal study cohort with paired biopsies, mean PRO-C3 increased with worsening of fibrosis and decreased with fibrosis improvement. The results suggest that PRO-C3 may be a potentially useful biomarker in identifying patients with advanced fibrosis and active fibrogenesis, as well as in assessing changes in fibrosis over time. It is worthy of further evaluation to confirm its diagnostic value and clinical utility.

## Introduction

Nonalcoholic fatty liver disease (NAFLD) affects over a quarter of adults in North America^1^. It has two principal phenotypes: nonalcoholic fatty liver (NAFL) or simple steatosis and nonalcoholic steatohepatitis (NASH). NASH is increasing in prevalence as an etiology for end-stage liver disease and also hepatocellular carcinoma, and is anticipated to increase the need for liver transplantation^2^, particularly as there is no currently approved therapy for NASH.

A key component of the assessment of NASH is the determination of the extent of liver fibrosis, which has been linked to the risk of mortality and liver-related clinical outcomes^3,4^. NASH patients with fibrosis stage 2 or greater, especially those with advanced fibrosis (stage 3–4), are at increased risk of death^3^. A major barrier to identify this subset of patients is the need for a liver biopsy to define the presence of NASH with moderate-advanced fibrosis (stage 2–3). Liver biopsies are invasive and occasionally associated with severe morbidity^5^. Moreover, interpretation of liver biopsy sections is limited by sampling variability and both intra- and inter-observer variability in assessment^6,7^. These carry the potential harm to patients and are also a barrier to recruitment to clinical trials. There is thus an urgent need to develop non-invasive tools for the assessment of the liver fibrosis in those with suspected NAFLD.

Few non-invasive biomarkers indirectly associated with fibrosis stages in NASH, such as FIB-4, aspartate aminotransferase (AST)/Alanine aminotransferase (ALT) ratio and NAFLD Fibrosis Score (NFS), have been extensively studied^8–11^. Recently, there has been growing interest in interrogating the biomarkers that directly reflect the process of fibrogenesis and fibrolysis to fully understand the dynamic nature of fibrosis. N-terminal pro-peptide of type III collagen (PIIINP) has been reported as a biomarker of tissue repair and liver fibrosis in a number of liver diseases^12–15^. However, a number of different PIIINP assays have been used in these studies and the antibodies used in these assays are not specific to the epitope at the cleavage site, thus the measured PIIINP may not truly reflect the levels of the cleaved pro-peptide. The quantification of circulating collagen fragments with unique epitopes released during collagen formation and degradation may have value in determining the extent and direction of extracellular matrix (ECM) turnover. A number of collagen fragments reflecting collagen formation, including PRO-C3 (N-terminal pro-collagen III peptide), PRO-C5 (C-terminal pro-peptide of type V collagen), PRO-C6 (C-terminal pro-peptide of type VI collagen) and basement membrane biomarker P4NP7S (7 S domain of type IV collagen), and those representing collagen degradation including C3M (Matrix Metalloprotease (MMP) degradation fragment of collagen III) and C4M (MMP degradation fragment of collagen IV), have been evaluated in preclinical models and patients^16–18^. Unlike the case of PIIINP, the PRO-C3 assay developed by Nordic Bioscience uses an antibody specifically recognizing the neo-epitope at the cleavage site, therefore PRO-C3 may be more reflective of the synthesis of type III collagen and fibrogenesis^19^. Patients with chronic hepatitis C (CHC) who had higher baseline PRO-C3 levels (≥20.2 ng/ml) showed progression (worsening) of fibrosis while those with lower PRO-C3 did not progress^19,20^. Further, PRO-C3, PRO-C5 and C4M have also been correlated with hepatic venous pressure gradient which is an invasive diagnostic and prognostic marker in cirrhosis for portal hypertension^21,22^. However, these collagen fragments have not been evaluated in patients with NASH.

The current study represents an initial assessment of a panel of collagen neo-epitope biomarkers (PRO-C3, P4NP7S, PRO-C5, PRO-C6, C3M, and C4M) to determine their relationship to fibrosis stage in a population of subjects with NAFLD and the full spectrum of fibrosis. The study included both a cross-sectional cohort and a longitudinal cohort with blood samples obtained at the time of liver biopsy.

## Results

### Characteristics of the study population for cross sectional analysis

Twenty-three patients with simple steatosis (NAFL) and 141 patients with NASH were included in this study as a discovery cohort. The NASH cohort had fibrosis stages from F0 to F4 (Table 1) and NAS from 1–7. The study cohort had a mean age of 53.2 years and comprised mostly of females (67.7%). Clinical characteristics, including albumin, alkaline phosphatase (ALP), aspartate aminotransferase (AST), Alanine aminotransferase (ALT), bilirubin and platelets, were available for a majority of the patients. Patients with F3-4 fibrosis tended to be older, had lower albumin and platelets, higher bilirubin, ALP and AST. However, there was no difference in ALT among the groups.

**Table 1:** Clinical and demographic characteristics for NAFLD patients by fibrosis stages.

	NAFL	NASH					
	F0	F0	F1	F2	F3	F4	P-value
	(N = 23)	(N = 16)	(N = 24)	(N = 41)	(N = 31)	(N = 29)	
Gender (M/F)	7/16	5/11	10/14	14/27	10/21	7/22	0.7507^a^
Age [median (Q1, Q3)]	55.6 (47.3,58.7)	43.7 (37.6,54.6)	47.5 (43.4,56.3)	54.5 (48.5,60.0)	57.1 (47.5,61.4)	57.0 (50.9,64.5)	0.0116
ALT (U/L) [median (Q1, Q3; N)]	57.5 (40.8,69.8;22)	66 (34,90;15)	59.5 (43.5,74.3;20)	51 (39,95;37)	71 (37.5,108;23)	45.5 (40,55;26)	0.3951
AST (U/L) [median (Q1, Q3; N)]	42.5 (32,51;22)	41 (29.5,45.5;15)	41.5 (34,52.8;20)	44 (31,62;37)	59 (39.5,75.5;23)	50.5 (37.8,63.3;26)	0.0335
ALP (U/L) [median (Q1, Q3; N)]	93.5 (77.5,113.5;22)	86 (66,107;15)	88 (79,102;20)	88 (77,106;37)	103 (87.5,135.5;23)	109.5 (98.3,148.5;26)	0.0253
Total Bilirubin (mg/dl) [median (Q1, Q3; N)]	0.5 (0.4,0.6;21)	0.5 (0.35,0.6;15)	0.5 (0.4,0.5;20)	0.6 (0.4,0.7;37)	0.6 (0.5,0.9;23)	0.8 (0.58,1.3;24)	0.0046
Albumin (g/dl) 1 [median (Q1, Q3; N)]	4.4 (4.3,4.6;22)	4.5 (4.4,4.7;15)	4.5 (4.3,4.7;20)	4.5 (4.2,4.6;37)	4.3 (4.2,4.5;23)	4.1 (3.8,4.4;26)	0.0021
Platelet Count (x10*9 c/L) [median (Q1, Q3; N)]	292.5 (218.8,344.5;10)	279 (232,288;7)	267 (210,298.5;7)	231 (187.3,290.8;12)	199 (180,226.8;14)	193.5 (132.8,211;8)	0.0485

^a^Chi-square test for sex, Kruskal-Wallis test for other parameters.

### Serum collagen fragment levels and association with histological scores in NAFLD

**Figure 1:**
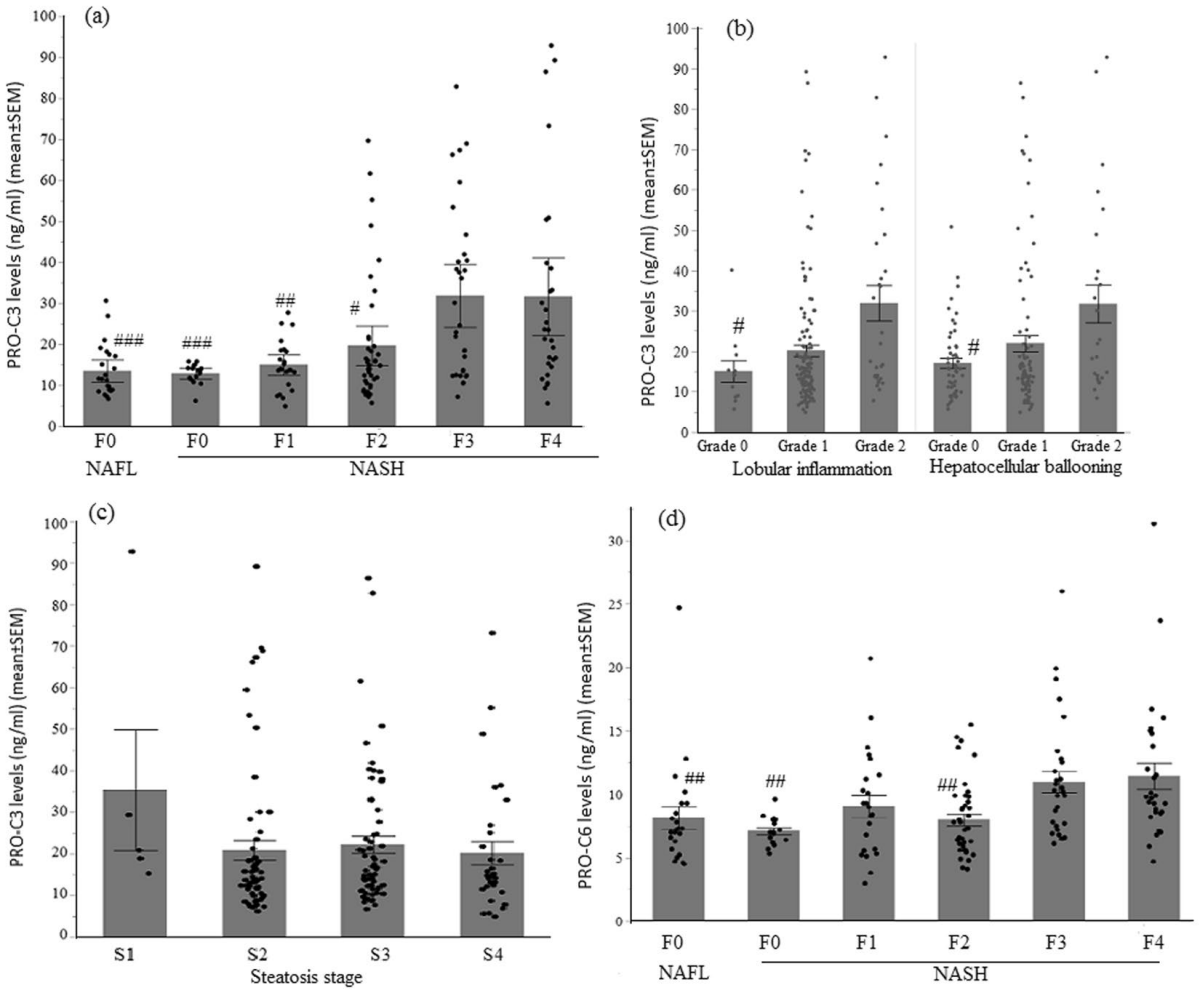
Association of PRO-C3 and PRO-C6 with histological scores. (**a**) Serum PRO-C3 levels were significantly higher in patients with advanced fibrosis (F3–4). ^###^p < 0.002 compared to F3 or F4; ^##^p < 0.006 compared to F3 or F4; ^#^p < 0.008 compared F3 or F4. (**b**) Elevation of PRO-C3 in patients with severe lobular inflammation and hepatocellular ballooning grade. ^#^p < 0.02 compared to Grade 2. (**c**) Serum PRO-C3 levels do not correlate with steatosis grade. (**d**) Serum PRO-C6 levels were significantly higher in patients with advanced fibrosis. ^##^p < 0.004 compared to F3 or F4. Means with standard error were displayed. Non-parametric Wilcoxon analysis were performed to compare each group.

In this cohort of NAFLD patients, PRO-C3 and PRO-C6 levels were correlated to each other (R = 0.57, p < 0.0001)), while the levels of C3M, C4M, P4NP7S and PRO-C5 were highly correlated with each other (Figure S1). All collagen biomarkers showed significant association with fibrosis stages (Table 2) with p < 0.0001 for both PRO-C3 and PRO-C6. In particular, PRO-C3 levels were significantly higher in patients with F3 or F4 than those with F0-F2 (Fig. 1a). In addition, PRO-C3 levels were also significantly higher in patients with severe lobular inflammation or hepatocellular ballooning degeneration (Fig. 1b). However, PRO-C3 levels were not associated with steatosis grade (Fig. 1c). Although PRO-C6 levels were significantly associated with fibrosis stages, it was only significantly higher in patients with F3-4 when compared to those with F0 or F2, but not with F1 due to variability in the latter group (Fig. 1d). Unlike PRO-C3, PRO-C6 had no correlation with lobular inflammation or ballooning although they both showed no association with or steatosis (Figure S2a–c). Median PRO-C3 levels in F3-4 (23.6 ng/ml) were 70% higher than that in F0-2 (13.9 ng/ml), while median PRO-C6 levels in F3-4 (9.9 ng/ml) were only 26% higher than that in F0-2 (7.3 ng/ml). Neither PRO-C3 nor PRO-C6 was able to differentiate F4 from F3. Higher levels of C4M, C3M and P4NP7S were only observed when comparing F3-4 with F0 or F2, but not with F1 possibly due to greater variability in the F1 group (Figure S3). PRO-C5 did not show consistent elevation in F3 and F4. None of the collagen biomarkers showed significant difference between F2 and F0-1.

**Table 2:** Serum collagen biomarker levels by fibrosis stages.

	NAFL	NASH					P-value (all stages)	P-value (F3–4 vs F0–2)
	F0	F0	F1	F2	F3	F4		
	(N = 23)	(N = 16)	(N = 24)	(N = 41)	(N = 31)	(N = 29)		
PRO-C3	11.5 (8.9,17.3)	13.5 (11.7,14.4)	14 (12.5,18.5)	14.8 (10.5,19.5)	24.6 (13,41.2)	23.5 (15.3,38.5)	<0.0001	<0.0001
PRO-C6	7.2 (6,8.9)	7 (6.4,8)	8.7 (5.6,11.3)	7.3 (5.9,9.4)	10.2 (7.6,12.2)	9.7 (8.6,13.8)	<0.0001	<0.0001
C4M	17.3 (14.9,23.7)	15.2 (13,17.7)	19.2 (15.7,21.8)	16.5 (14.5,19.7)	22.6 (16.5,24.7)	22 (15.6,28.9)	0.001	0.003
C3M	7.2 (6,8.95)	6.4 (5.3,7.55)	8.2 (6.8,10.25)	7.5 (6.3,8.5)	8.4 (7.15,11.35)	8.2 (6.6,9.9)	0.005	0.006
P4NP7S	110.5 (97,146.9)	107 (85.9,125)	124.8 (107.7,153.7)	111.7 (90.9,148.7)	151.2 (113.6,173.3)	137.4 (116.4,174.8)	0.008	0.007
PRO-C5	270.9 (204.2,345.2)	202.8 (168.5,267.8)	285.3 (191.1,401.5)	268.8 (212.7,368.2)	345.1 (236.9,511.5)	262.5 (185.1,462.6)	0.020	0.025

Values are median (ng/ml) (Q1, Q3). P values were generated by Kruskal-Wallis analysis.

### Performance of serum collagen fragments to discriminate fibrosis stages in NAFLD

Univariate analysis demonstrated that high levels of all measured collagen biomarkers were risk factors for advanced fibrosis (F3-4) (Table 3). However, in multivariate analysis adjusting for available clinical characteristics, among collagen fragments only PRO-C3 and PRO-C6 remained significantly and independently related to advanced fibrosis (Table 3). They were further evaluated by performing Receiver operating characteristic curve (ROC) analysis (Table S1 and Figure S4). PRO-C3 and PRO-C6 had the highest AUROC (Area under receiver operating characteristic curve) (0.74 for PRO-C3 and 0.73 for PRO-C6) to differentiate F3-4 from F0-2. Combination of PRO-C3 and PRO-C6 only slightly improved performance to identify F3-4 (AUROC 0.77). A cutoff of 20.9 ng/ml for PRO-C3 was obtained to discriminate advanced fibrosis using ROC Youden index, which is very close to the cutoff (20.2 ng/ml) reported in patients with hepatitis C^19^. By applying the cutoff of ≥20.9 ng/ml in this NAFLD cohort, PRO-C3 could identify patients with advanced fibrosis (F3-4) with sensitivity of 57% and specificity of 84%, and identify patients with significant fibrosis (F2-4) with sensitivity of 46% and specificity of 90%.

**Table 3:** Univariate and multivariate analysis to identify biomarkers associated with advanced fibrosis.

	Univariate Analysis	p-value	Multivariate Analysis	p-value
	Advanced fibrosis (F3-4 vs F0-2) (OR 95% CI, N)		Advanced fibrosis (F3-4 vs F0-2) Odds Ratio (95% CI, N)	
PRO-C3	3.23 (2.06, 5.06; 165)	<0.0001	**1.84 (1.05, 3.23))**	**0.0341**
PRO-C6	5.01 (2.47, 10.16; 158)	<0.0001	**3.43 (1.33, 8.87)**	**0.0108**
C4M	4.42 (2.01, 9.71; 163)	0.0002		
P4NP7S	3.66 (1.77, 7.57; 163)	0.0005		
Alkaline Phosphatase	3.31 (1.58, 6.94; 141)	0.0015	**3.22 (1.29, 8.00)**	**0.0120**
Albumin	0.19 (0.07, 0.53; 141)	0.0016		
C3M	3.84 (1.64, 9.00; 162)	0.0019		
Platelet	0.99 (0.98–0.99; 100)	0.0021		
AST	2.15 (1.25, 3.69; 141)	0.0057		
Age	1.05 (1.01, 1.08; 162)	0.0062	**1.06 (1.01, 1.11)**	**0.0185**
PRO-C5	1.82 (1.178, 2.808; 162)	0.007		
Total Bilirubin	1.67 (1.06, 2.62; 141)	0.0272		
Sex (Female 0, Male 1; N)	0.73 (0.37–1.46; 163)	0.3788		
ALT	0.87 (0.56, 1.35; 141)	0.5349		

*Complete cases, N = 136. Model selected using forward selection by best prediction of leave one out cross validation data (based on AUC); OR: Odds Ratio.

The performance of PRO-C3 and PRO-6 to identify patients with advanced fibrosis was further validated in an independent cohort. The baseline data set from a longitudinal cohort of 41 patients with baseline fibrosis stage from 1–3 was used for an independent validation (Table S2). In this cohort, only PRO-C3 levels were significantly associated with fibrosis stages (Table S3). The AUROC of PRO-C3 to discriminate patients with advanced fibrosis from those with F1-2 was approximately 0.75 (Figure S5), which is very similar to the AUROC in the discovery cohort (Table S1). Similar to the performance in the discovery cohort, the cutoff of 20.9 ng/ml identified in the discovery cohort can identify patients with advanced fibrosis in the validation cohort with a sensitivity of 57% and specificity of 79%.

We further explored the performance of simple scores such as FIB-4 in this cohort. FIB-4 data were only available in a subset of patients (n = 96). In this sub-cohort, FIB-4 and PRO-C3 levels were moderately correlated (Spearman correlation with R^2^ = 0.29, p < 0.0001) (Figure S6). FIB-4 had an AUROC of 0.82 to discriminate patients with advanced fibrosis from those with F0-2. PRO-C3 had AUROC of 0.74, which is very similar to the performance in the whole cohort.

### Association of longitudinal changes of PRO-C3 with changes in fibrosis stage

A longitudinal cohort of 41 patients with baseline and follow up biopsies were identified to explore whether the collagen biomarkers are related to fibrosis progression/regression. The patients were followed up for an average of 3.5 years ranging from 1–10 years. The status of fibrosis stages of these patients at baseline and follow up visits are shown in Table 4. Nine patients had progression/worsening (≥1 stage increase in fibrosis), fifteen patients had regression/improvement (≤1 stage decrease in fibrosis) and sixteen patients had no change. Baseline clinical characteristics and average follow up durations were not significantly different among the three groups (Table 5). Among all collagen biomarkers measured, only PRO-C3 levels were significantly higher in patients with advanced fibrosis (F3) at both baseline and follow up visits (Table S3, Figure S7).

**Table 4:** Fibrosis stage changes from baseline at follow up visit.

Baseline fibrosis stages	Follow up fibrosis stages (number of patients)			
	F0	F1	F2	F3
F1 (n = 19)	**4**	10	**2**	**3**
F2 (n = 15)	**2**	**6**	2	**5**
F3 (n = 7)	**1**		**2**	4

**Table 5:** Baseline characteristics for a cohort of NASH patients with biopsies at baseline and follow up.

	All patients	Fibrosis Improvement	Fibrosis no Change	Fibrosis worsening	P value
Age	50.1 ± 1.7 (41)	50.8 ± 2.8 (15)	49 ± 2.72 (16)	50.5 ± 3.5 (10)	0.886
Gender M/F (%)	13/41 ((32%)	6/15 (40%)	5/16 (31%)	2/10 (20%)	0.631
Diabetes (%)	15/41 (37%)	3/15 (20%)	8/16 (50%)	4/10 (40%)	0.228
AST (U/L) (N)	71.3 ± 8.1 (39)	68.8 ± 9.9 (15)	69.4 ± 17.47 (15)	78.4 ± 12.9 (9)	0.893
ALT (U/L) (N)	98.3 ± 9.2 (39)	97.8 ± 11.0 (15)	97.5 ± 10.0 (15)	100.3 ± 10.9 (9)	0.993
ALP (U/L) (N)	97.4 ± 4.4 (39)	90.4 ± 7.7 (15)	95.9 ± 6.2 (15)	111.2 ± 9.3 (9)	0.205
total bilirubin (mg/dl) (N)	0.58 ± 0.04 (40)	0.74 ± 0.08 (15)	0.48 ± 0.05 (15)	0.51 ± 0.08 (9)	0.038
Albumin (g/dl) (N)	4.6 ± 0.0 (40)	4.6 ± 0.1 (15)	4.5 ± 0.1 (15)	4.6 ± 0.1 (9)	0.867
Fibrosis stage (N)	1.7 ± 0.2 (41)	1.9 ± 0.2 (15)	1.6 ± 0.2 (16)	1.5 ± 0.2 (10)	0.321
Steatosis grade (N)	1.8 ± 0.1 (41)	1.8 ± 0.2 (15)	1.8 ± 0.1 (16)	1.9 ± 0.3 (10)	0.897
Lobular inflammation grade (N)	1.2 ± 0.1 (41)	1.3 ± 0.2 (15)	1.1 ± 0.1 (16)	1.2 ± 0.1 (10)	0.581
Hepatocellular Ballooning grade (N)	1.2 ± 0.2 (41)	1.1 ± 0.2 (15)	1.1 ± 0.2 (16)	1.1 ± 0.2 (10)	0.867
biopsy interval (year) (N)	3.4 ± 0.3 (41)	3.5 ± 0.5 (15)	3.2 ± 0.4 (16)	3.5 ± 0.4 (10)	0.878

Values are Mean ± standard error of mean. p values were generated by Anova analysis.

**Figure 2:**
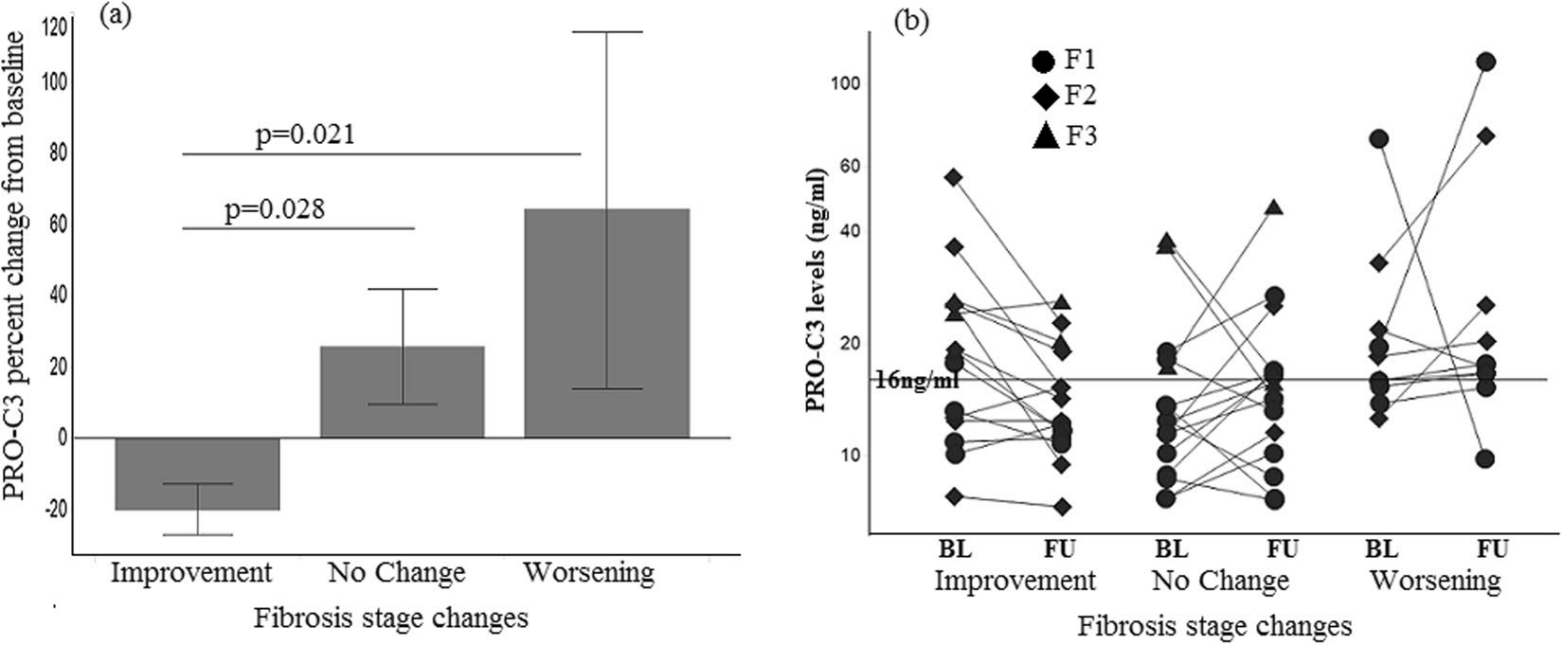
Association of PRO-C3 change from baseline with fibrosis stage changes. (**a**) Patients with fibrosis improvement during follow up had significant decrease of PRO-C3 from baseline compared to those with worsening or stable fibrosis. Mean percent changes from baseline with standard error were shown. Non-parametric Wilcoxon analysis were performed to compare each group. (**b**) PRO-C3 changes from baseline at follow up for individual patients. BL: baseline; FU: follow up.

We evaluated whether the changes of collagen biomarkers were associated with changes in fibrosis stage. The mean percent change of PRO-C3 in the group showing improvement (or regression) in fibrosis was approximately −20%, which is significantly lower than that in the groups with stable (25%) or worsening (64%) fibrosis (Fig. 2a). The changes of PRO-C3 for individual patients by fibrosis stage change are shown in Fig. 2b. For the patients with improvement in fibrosis, 9 out of 15 patients (60%) had a decrease of PRO-C3 by more than 15% (an arbitrary cutoff based on the reported 10% inter-assay variability)^23^. Interestingly, 8 out of the 9 patients with ≥15% decrease of PRO-C3 in the fibrosis improvement/regression group had baseline PRO-C3 levels greater than 16 ng/ml (Fig. 2b), which is the median baseline levels of PRO-C3 in this cohort. However, 5 of the 6 patients with baseline PRO-C3 levels lower than 16 ng/ml in the same group had <15% decrease of PRO-C3 (Fig. 2b).

The changes in other collagen biomarkers were not significantly different when comparing the group with improved fibrosis to that with worsening fibrosis, although greater increase in these biomarkers were observed in the group with stable fibrosis than that with improved fibrosis (Figure S8).

## Discussion

To our knowledge, the current study represents the first to assess the diagnostic performance of circulating collagen fragments reflective of fibrogenesis or fibrolysis to determine the fibrosis stage in patients with NAFLD. Elevated PRO-C3 levels were significantly associated with advanced fibrosis stage in both discovery and validation cohort. In addition, higher PRO-C3 levels were associated with higher grade of lobular inflammation and ballooning, suggesting that PRO-C3 levels may be reflective of active disease. Furthermore, decrease of PRO-C3 over time was associated with improvement (or regression) in fibrosis.

A ROC curve is an established method for evaluating the clinical validity of a biomarker. In this study, AUROC for PRO-C3 was approximately 0.74, which is much lower than the AUROCs reported for transient elastography and Magnetic Resonance Elastography (MRE)^24^ and is also lower than FIB-4 in a sub-cohort in this study. However, the relevance of ROC is linked to the accuracy of the reference standard (which in this case is liver biopsy) and its interpretation. Even in excellent hands, liver biopsy can misclassify fibrosis stage in up to 20% of subjects^6,7^. In addition, ROC weigh false positives and false negatives equally and do not provide information on the predictive values needed when used clinically. Furthermore, unlike elastography which measures liver stiffness, PRO-C3 measures active collagen formation reflective of fibrogenesis, but not exactly the fibrosis area in the liver. This may be one of the reasons to explain the lower performance of PRO-C3 than MRE in identifying fibrosis measured by histology. The cutoff of ≥20.9 ng/ml for PRO-C3 derived from ROC analysis had low sensitivity (57%) and moderate specificity (79%), suggesting that nearly 40% of the cases with advanced fibrosis would be missed while nearly 20% of the cases may be misclassified. The modest performance of PRO-C3 in discriminating advanced fibrosis suggest that PRO-C3 as a single marker is sub-optimal for diagnosing static fibrosis stages. However, a combination of PRO-C3 with other biomarkers reflecting underlying pathophysiology of the disease may improve the diagnostic performance. Again, our data suggest that PRO-C3 may be more valuable for identifying patients with active fibrogenesis than diagnosing static disease stages.

Some patients with advanced fibrosis may have less active disease with old scars, and thus may have low levels of PRO-C3. This may explain the normal levels of PRO-C3 in some F3-4 patients. One would predict that such patients would have a lower risk of fibrosis progression if PRO-C3 levels stay low. This is supported by the data that patients with chronic hepatitis C having higher PRO-C3 demonstrated a higher rate of fibrosis progression than those having lower PRO-C3^19^. Interestingly, for patients showing improvement in fibrosis in the longitudinal cohort, PRO-C3 decreased over time in those with higher baseline PRO-C3 levels (>16 ng/ml), suggesting that fibrosis in patients with active tissue remodeling (as reflected by higher PRO-C3 levels), would regress if PRO-C3 levels were decreased, possibly resulting from the removal or reduction of the insults causing tissue injury. If there were repetitive insults, persistent tissue remodeling as reflected by sustained elevation of PRO-C3, would lead to fibrosis progression over time. Although in this small study cohort, both stable and progressive fibrosis groups had an increase in mean PRO-C3, it is possible some patients in the stable group may progress if the follow up time were extended. Although FIB-4 had better diagnostic performance for fibrosis staging, change in FIB-4 at follow up had no correlation with changes in fibrosis stages in this study cohort (data not shown), indicating that PRO-C3 may be a more sensitive biomarker to monitor disease progression/regression.

One patient who showed marked decrease of PRO-C3 (from 71 to 9.8 ng/ml) (Fig. 2b) in the fibrosis progression group also had a decrease in ALT (from 160 to 47 U/L) and AST (from 132 to 51 U/L). This patient progressed from F1 to F3 and had only grade 1 steatosis, inflammation and ballooning at baseline and follow-up. The duration between baseline and follow up biopsies was approximately four years. It is not known how long PRO-C3 and ALT had remained elevated after baseline visit and when they started to decline. It is possible that the patient may have progressed to F3 and stayed stable before the follow up visit. Unfortunately serum samples were not available between baseline and follow up to evaluate the kinetics for PRO-C3 change. We cannot exclude the possibility that this particular patient may have acute liver injury on top of NASH to drive the disease progression.

One key consideration in the assessment of any biomarker is the biological plausibility of its relationship to the pathological or physiological condition it measures. The use of collagen fragments for the assessment of fibrotic disorders is now well established^16,18,19,22,23,25,26^. Type III collagen, one of the main components in interstitial matrix, is secreted by fibroblasts and other mesenchymal cells, playing an important role in inflammation-fibrosis associated pathologies in tissue injury^27^. PIIINP, measured by a number of assays, has been reported as a marker of tissue repair and liver fibrosis in some liver diseases^12–15^. PIIINP is also a component of Enhanced Liver Fibrosis (ELF), a calculated index consisting of PIIINP, hyaluronic acid and tissue inhibitor of matrix metalloproteinase 1(TIMP 1)^28^. ELF has demonstrated variable performance (AUROC 0.76–0.9) in identifying NASH patients with advanced fibrosis^10,15,29^. Tanwar *et al*. reported that PIIINP levels measured in ELF test were significantly elevated in a cohort of NAFLD patients with advanced fibrosis compared to those with F0-2, and had an AUROC of 0.85 to distinguish simple steatosis vs NASH or F3-4^15^, however, the study did not specifically compare F0-2 vs F3-4 in NASH patients. It is worthwhile to compare the performance of PIIINP and PRO-C3 in future studies. It should be pointed out that different assays, such as ELISA^30,31^, radioimmunoassay (RIA)^12,13^ and immunoassays using magnetic particle separation technique^15,32,33^, or automated ADVIA Centaur immunoassay^34^, have been used to measure serum “PIIINP” in reported studies. It has been demonstrated that there are almost two fold difference in the measured levels of PIIINP when UniQ RIA assay, which has been widely used, was compared with the new ADVIA Centaur immunoassay which has been tested as a component in ELF test by Siemens Healthcare Diagnostic^34^. It is almost impossible to evaluate PIIINP levels across studies due to the lack of standardized assay. PIIINP antibodies employed in these assays were not disclosed and may not be specific to the pro-peptide cleavage site, thus the measured PIIINP may not truly reflect cleaved pro-peptide. The PRO-C3 assay uses an antibody specifically recognizing the neo-epitope that is exposed upon cleavage, therefore PRO-C3 may be more reflective of the synthesis of type III collagen and fibrogenesis^19^. It is interesting also to note that, in the our cross sectional cohort, PRO-C3 levels were significantly related to lobular inflammation and hepatocellular ballooning providing more evidence to the concept that disease activity and fibrogenesis are biologically related.

PRO-C6, which is the C-terminal pro-peptide of collagen VI and is highly expressed in adipose tissue, is a newly identified adipokine called endotrophin^35^. Endotrophin has been shown to promote adipose tissue fibrosis^36^. This is the first study showing the correlation of PRO-C6 with PRO-C3 and liver fibrosis. Although the association with fibrosis was not significant in the small validation cohort, a trend of higher PRO-C6 was observed in F3 (p = 0.07, table S3). It is possible that the association of PRO-C6 with liver fibrosis is indirect and is attributed to the link of adipose fibrosis and liver damage^37^.

There are at least two specific contexts of application where PRO-C3 could be used: to identify patients with active fibrogenesis and advanced fibrosis (stage 3 and 4); to monitor the changes in fibrosis stage over time. The small data set presented in this analysis demonstrates that changes in PRO-C3 may distinguish between those who regressed versus those who progressed. But the failure to separate those with stable disease from those who progressed may be due to the small sample size and heterogeneous baseline fibrosis stages, hence the need for continued efforts to optimize the use of this and other biomarkers for this purpose.

There are several limitations for this study. One major limitation is that this is a retrospective analysis of samples collected over a long period of time (up to 10 years). Even though the samples were stored in −80 °C, the stability of the biomarkers over this long period has not been tested. The baseline visits from longitudinal cohort was used to validate the performance of PRO-C3, and this cohort is enriched with patients with F1-2 stages, which is not an ideal validation cohort. Unfortunately, metabolic parameters (such as HbA1c, insulin resistance, triglyceride) that are high risk factors for NASH development were only tested in less than 20% of the patients at the time of biopsy and sample collection. Therefore we were not able to include these parameters in multivariate analysis. The longitudinal study cohort is very small and has heterogeneous baseline fibrosis stages and follow up duration, which limits further analysis. Majority of the patients in the stable fibrosis group had F1-2 at baseline and there was no F3 patient in the worsening group, which may lead to bias in analysis. The patients were enrolled as part of a study on the natural history of NAFLD, therefore we cannot exclude the possibility of potential confounding factors, such as comorbidities, medications, and diurnal or day to day changes that may affect the analysis.

In summary, the current study is the first to evaluate the association of collagen fragments with fibrosis stage in those with NAFLD. The data indicate that PRO-C3 may have potential utility to identify patients with active fibrogenesis and to monitor fibrosis changes in patient care or clinical trial. While much further work is needed before the clinical utility of PRO-C3 is established for NAFLD, the current studies are encouraging and provide a strong rationale for future studies to further validate PRO-C3 as a biomarker to diagnose and monitor liver fibrosis.

## Materials and Methods

This work represents an analysis of plasma samples obtained on the day of liver biopsies performed for the assessment of suspected or known NAFLD. These samples were obtained as part of a study on the natural history of NAFLD and with the aim to also evaluate circulating factors associated with changes in disease phenotype over time. The study was carried out in accordance with approved guidelines by the Institutional Review Boards at Virginia Commonwealth University (VCU IRB 1960) and all subjects provided informed consent.

### Study Populations

Two sets of subjects were studied as part of the project. The cross-sectional set included individuals with known or suspected NAFLD who were undergoing a standard of care liver biopsy to assess their liver histology and whose biopsy demonstrated the presence of NAFLD. This cohort was used as discovery cohort to evaluate the performance of biomarkers to differentiate fibrosis stages. A second independent longitudinal set included a group of individuals, on whom baseline liver biopsy and samples were available, who underwent a follow up liver biopsy for assessment of disease progression as part of standard of care. Baseline values from this cohort were used as an independent validation cohort to evaluate the performance of PRO-C3 and PRO-C6 to identify advanced fibrosis.

### Case Definitions

NAFLD was confirmed by histology in all cases. The nonalcoholic nature of the liver disease was assessed clinically from a history of consumption of alcohol below the thresholds commonly used (≤20 gm/day for women and ≤30 gm/day for men)^38^. Subjects were classified to have either NAFL (simple steatosis or steatosis with minimal inflammation) or NASH. For purposes of this analysis, both borderline and definite steatohepatitis were considered as steatohepatitis. The disease activity was scored using the NASH CRN NAFLD activity score (NAS)^39^. The fibrosis stage 0–4 was also classified using the NASH CRN fibrosis staging system. The sub-stages of stage 1 were combined as a single stage. All biopsies were read by hepato-pathologists (Michael Idowu and Melissa J Contos) who were masked to the results of the fibrosis markers. Where there were inconsistencies in the reading of a biopsy sample, both pathologists consulted with each other to give a common consensus opinion.

### Sample collection and analysis

Serum samples collected on the day of the liver biopsy (before the procedure) were stored at −80 °C within 1–2 hours of blood collection until laboratory analysis. Sample analysis was performed by Nordic Biosciences (Denmark) without access to any clinical or histological information associated with any sample using previously published immunoassays^21–23,25^. The inter-assay (plate-to-plate consistency) and intra-assay (repeat testing of the same sample) variabilities for these biomarkers were up to 12% and 5%, respectively^21^.

### Data collection and statistical analysis

Clinical data were extracted from medical records and stored in a de-identified data set in an electronic spreadsheet. Metabolic parameters including glucose, triglyceride, LDL and HDL were not tested for most of the patients at the visit for biopsy, therefore, were not available for the analysis. The histology was scored and entered in to a separate spreadsheet. A third spreadsheet with identifiers connecting each sample identifier to a given subject was maintained in a secure HIPAA-compliant institutional server behind appropriate firewalls. The clinical, histological and laboratory data were merged and sent to the statisticians with only a study ID number for each sample. Data analysis was performed by the statistician and fully reviewed by the investigators.

The non-parametric Wilcoxon test was used to compare the biomarker levels across fibrosis stages, the grade of steatosis, lobular inflammation and ballooning. An overall AUC (Area under curve) was constructed. In addition, the performance of PRO-C3 to distinguish F0-2 vs F3-4 was assessed using ROC Youden cutoff of 20.9 ng/ml and was further validated in an independent cohort.

Univariate and multivariate logistic regression models, assessing available clinical parameters and collagen fragment concentrations, were generated to relate to advanced fibrosis stage (Stage 3–4). Variable selection in multivariable model was conducted using forward stepwise selection and best prediction criteria, as determined by leave one out cross validation.

Complete case methods were used in all statistical assessments (missing data were not imputed). For the multivariable model, variables with at least 140 non-missing values were included. As a result, across all variables used in the model, there were 136 subjects with complete cases, compared to 164 subjects in the overall data set. Data were log transformed prior to logistic modeling, when appropriate. Statistical analyses were conducted using SAS 9.2 and R 3.2.2.

For the longitudinal cohort, pairwise comparisons were performed using the non-parametric Wilcoxon test.

## Electronic supplementary material

Supplementary Material

## Data Availability

The datasets generated during and/or analyzed during the current study are available from the corresponding author on reasonable request.
