# Supplementary material for: An Evaluation of the Collagen Fragments Related to Fibrogenesis and Fibrolysis in Nonalcoholic Steatohepatitis
Source: Sci Rep. 2018 Aug 17;8:12414. doi: 10.1038/s41598-018-30457-y (PMC6098042; doi:10.1038/s41598-018-30457-y)

Supplemental material

An Evaluation of the Collagen Fragments Related to Fibrogenesis and Fibrolysis in Nonalcoholic Steatohepatitis

Short title: PRO-C3 as a potential prognostic fibrosis biomarker in NASH

Yi Luo^1,#^, Abdul Oseini^2,#^, Robert Gagnon^1^, Edgar D Charles^1^, Kurex Sidik ^1^, Robert Vincent^2^, Rebeca Collen^2^, Michael Idowu^2^, Melissa J Contos^2^, Faridoddin Mirshahi^2^, Kalyani Daitya^2^, Amon Asgharpour^2^, Mohammed S. Siddiqui^2^, Gabor Jarai^1^, Glenn Rosen^1^, Rose Christian^1^ and Arun J. Sanyal^2^

Affiliations:

1. Fibrosis Translational Research and Development, Bristol-Myers Squibb, Pennington, NJ

2. Division of Gastroenterology, Hepatology and Nutrition, Virginia Common Wealth University, Richmond, VA

#Co-primary authors

Table S1: Performance of PRO-C3 and PRO-C6 to discriminate different fibrosis stages in NAFLD patients

|  | PRO-C3  AUROC | PRO-C6  AUROC | PRO-C3+PRO-C6 AUROC |
| --- | --- | --- | --- |
| F0 vsF1-4 | 0.70 | 0.67 | 0.71 |
| F0-1 vs F2-4 | 0.69 | 0.62 | 0.70 |
| **F0-2 vs F3-4** | **0.74** | **0.73** | **0.77** |
| F0-3 vs F4 | 0.68 | 0.69 | 0.70 |

Table S2. Clinical and demographic characteristics for validation cohort

|  | **F1** | **F2** | **F3** |  |
| --- | --- | --- | --- | --- |
|  | **(N=19)** | **(N=15)** | **(N=7)** | **P-value** |
| Gender (M/F) | 4/15 | 7/8 | 2/5 | 0.276 ^a^ |
| Diabetes (%) | 5/19 (26.3%) | 6/15 (40%) | 4/7 (57.1) | 0.331 ^a^ |
| Age  [median (Q1, Q3)] | 50.0  (41.3,56.6) | 51.0  (44.8,55.2) | 56.0  (46.0,59.5) | 0.566 |
| ALT (U/L)  [median (Q1, Q3; N)] | 83.5  (53.8,126.2;18) | 89.5  (62.5,122.5;14) | 90.0  (72,123;7) | 0.974 |
| AST (U/L)  [median (Q1, Q3; N)] | 54.0  (34.2,91.2;18) | 63.0  (38,94;14) | 70  (56,79.5;7) | 0.563 |
| ALP (U/L)  [median (Q1, Q3; N)] | 87.5  (82.5,107;18) | 101.5  (75.5,116.8;14) | 88  (78,100.5;7) | 0.720 |
| Total Bilirubin (mg/dl)  [median (Q1, Q3; N)] | 0.45  (0.32,0.5;18) | 0.7  (0.5,0.9;14) | 0.7  (0.45,0.8;7) | 0.044 |
| Albumin (g/dl) 1  [median (Q1, Q3; N)] | 4.5  (4.5,4.7;18) | 4.6  (4.3,4.9;14) | 4.6  (4.6,4.6;7) | 0.932 |
| Platelet Count  (x10*9 c/L)  [median (Q1, Q3; N)] | 294  (234,315;15) | 276  (236,318.8;8) | 225  (172,276;6) | 0.224 |

^a^ Chi-square test for sex, Kruskal-Wallis test for other parameters.

Table S3. Serum collagen biomarker levels by fibrosis stages in validation cohort

|  | **F1** | **F2** | **F3** |  |
| --- | --- | --- | --- | --- |
|  | **(N=19)** | **(N=15)** | **(N=7)** | **P-value** |
| PRO-C3 | 13.6  (10.6,16.8) | 18.6  (12.5,25.5) | 24.1  (18.3,31.4) | 0.038 |
| PRO-C6 | 6.8  (5.7,8.6) | 9.2  (6.2,12.2) | 9.3  (7.5,10.4) | 0.074 |
| P4NP7S | 113.9  (108.2,133.3) | 151.1  (105.6,171.6) | 111.5  (97.3,145.4) | 0.461 |
| PRO-C5 | 230.2  (191,264.1) | 260.4  (182.3,302.2) | 209.8  (199.6,232.8) | 0.507 |
| C4M | 20.6  (17.6,22.7) | 23.0  (17.0,33.5) | 22.7  (18.6,26.2) | 0.427 |
| C3M | 7.6  (6.4,8.4) | 7.8  (6.8,11.3) | 6  (5.7,7.5) | 0.117 |

Values are median (ng/ml) (Q1, Q3). P values were generated by Kruskal-Wallis analysis

Supplemental Figure Legends

FigureS1: Cross-Correlation of the serum collagen biomarkers. Values in the graphs are shown as Spearman’s rank correlation coefficients. All correlations shown are p<0.0001. Those with R>0.5 were highlighted. PRO-C3 levels were correlated with PRO-C6 levels. C3M, C4M, P4NP7S and PRO-C5 levels were more correlated with each other.

Figure S2: Serum PRO-C6 levels were not correlated with lobular inflammation (a), ballooning (b) or steatosis (c) grade in this cohort. Non-parametric Wilcoxon analysis were performed to compare each group. Means with standard error were displayed.

Figure S3: Correlation of the serum levels of collagen fragments with fibrosis stages. (a) P4NP7S. (b) PRO-C5. (c) C3M. (d) C4M. Non-parametric Wilcoxon analysis were performed to compare each group. The line indicated with comparison pairs. *p≤0.05. Means with standard error are displayed.

Figure S4: Receiver Operating Characteristic curve of PROC3 and PRO-C6 in discriminating patients with advanced fibrosis (F3-F4) from those with F0-2.

Figure S5: Receiver Operating Characteristic curve of PROC3 (AUROC 0.75) in discriminating patients with advanced fibrosis (F3) from those with F1-2 in validation cohort.

Figure S6: Correlation of FIB-4 and PRO-C3 in a subset of cohort (n=96). Spearman correlation analysis was performed. R^2^=0.29, p<0.0001.

Figure S7: (a) Association of PRO-C3 with fibrosis stages at baseline in the longitudinal cohort. Mean PRO-C3 levels were higher in patients with advanced fibrosis (F3) than those with F1. (b) Association of PRO-C3 with fibrosis stages at follow up visit. Mean PRO-C3 levels were higher in patients with F3 than those with F0 or F1. Non-parametric Wilcoxon analysis were performed to compare each group.

Figure S8: Changes of collagen biomarkers grouped by improved, stable and worsening fibrosis. Mean percent changes from baseline with standard error were shown. Non-parametric Wilcoxon analysis were performed to compare each group.

Figure S1


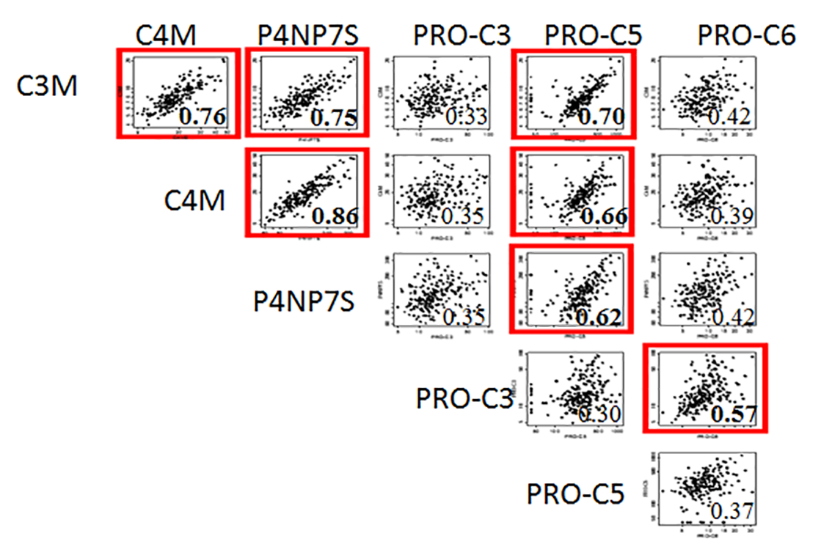


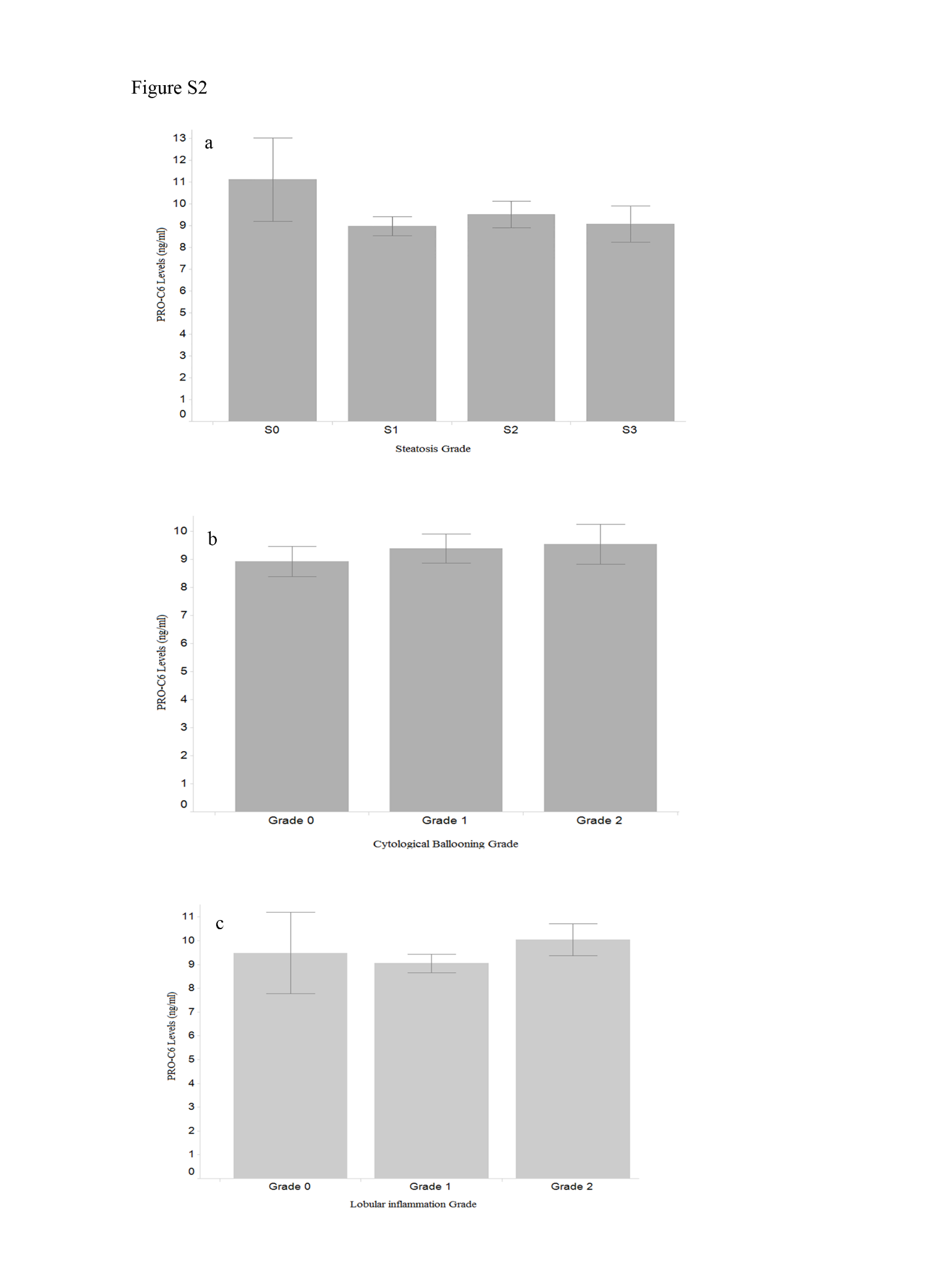


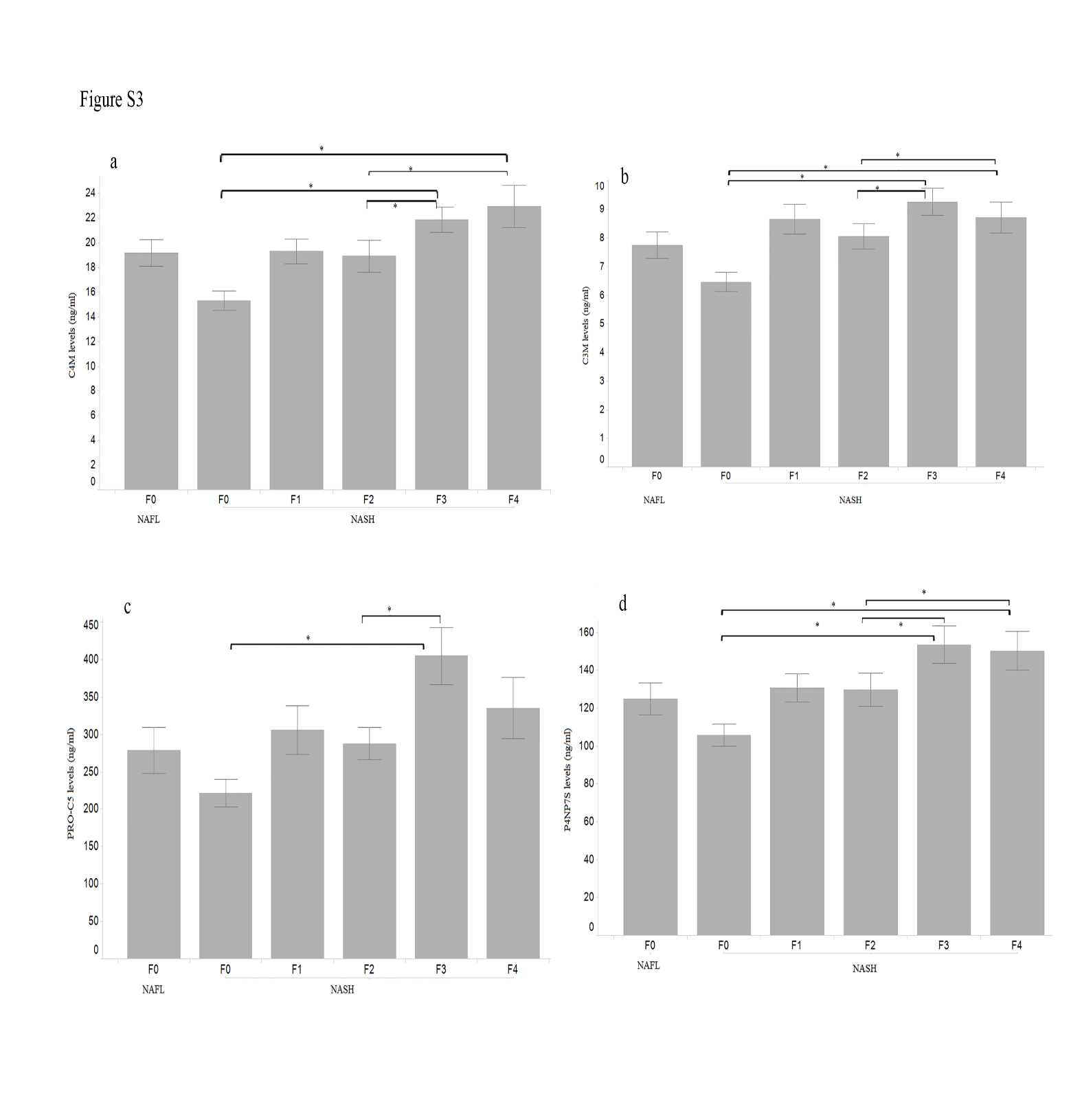


Figure S4


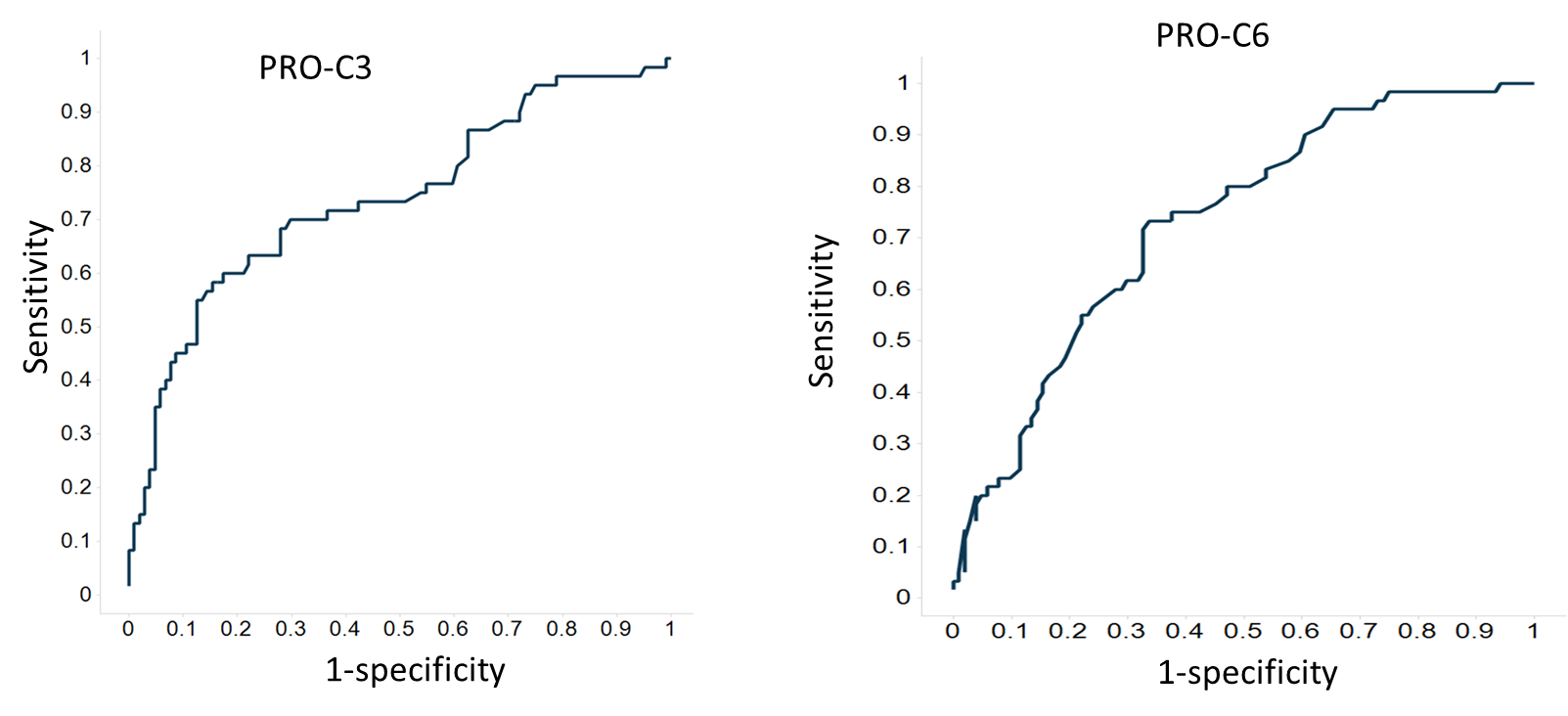


Figure S5


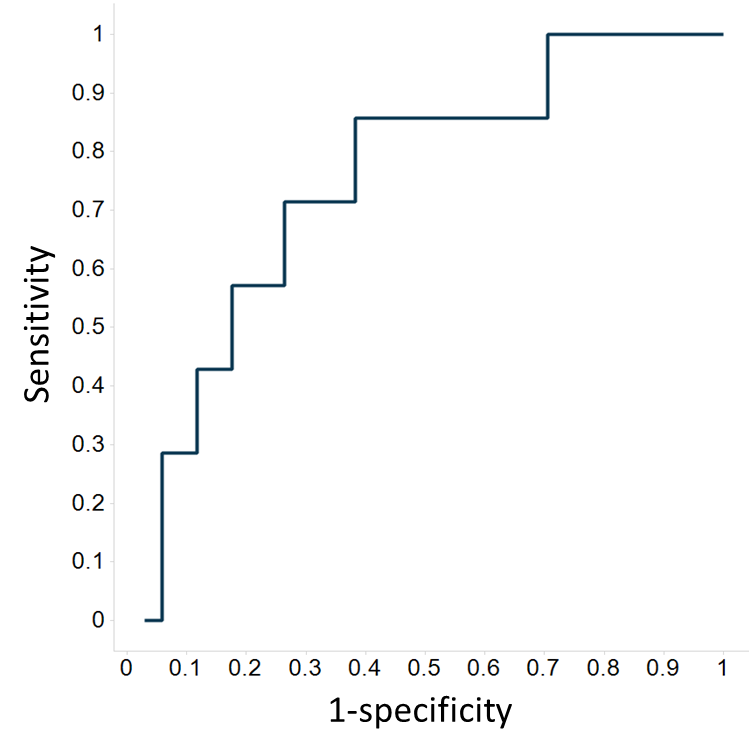


Figure S6


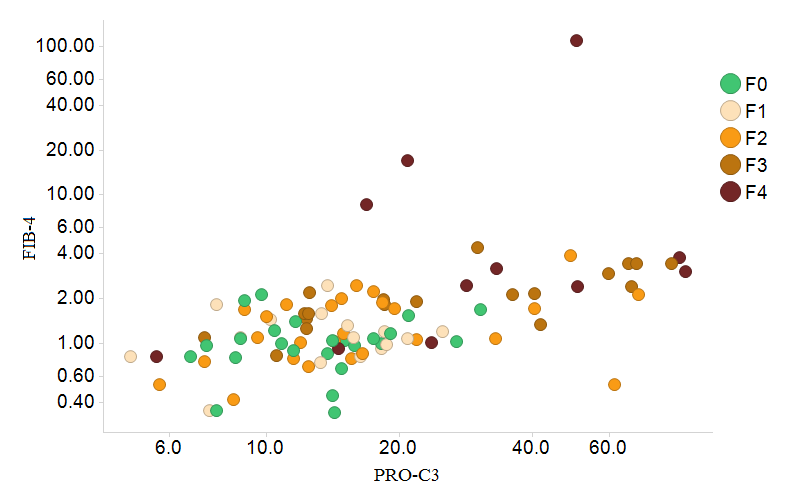


Figure S7


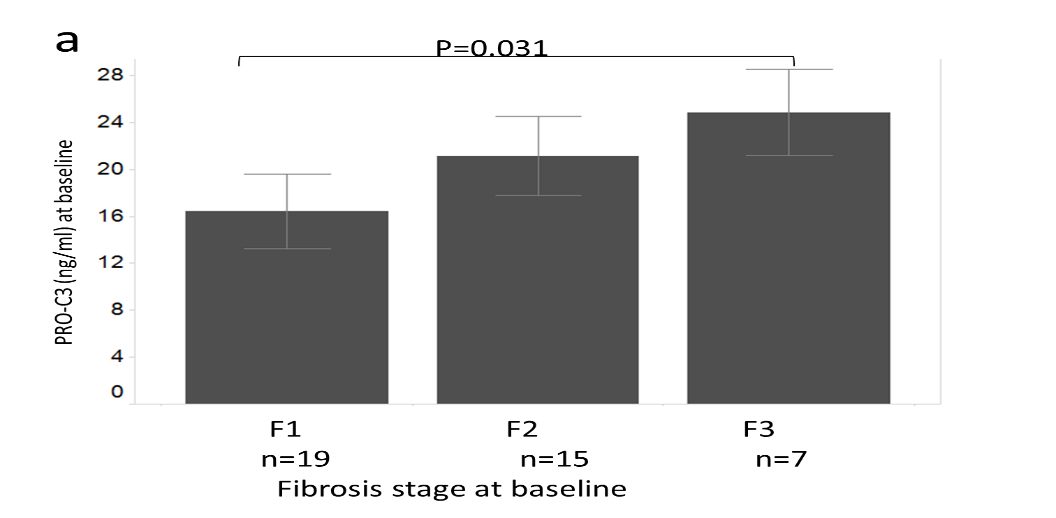


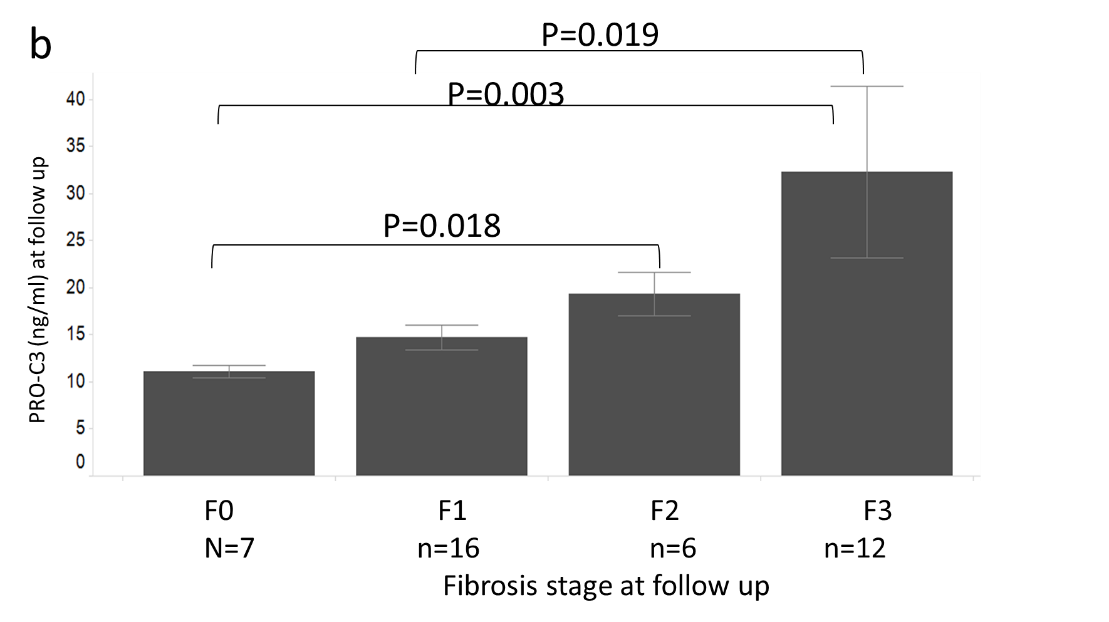


Figure S8


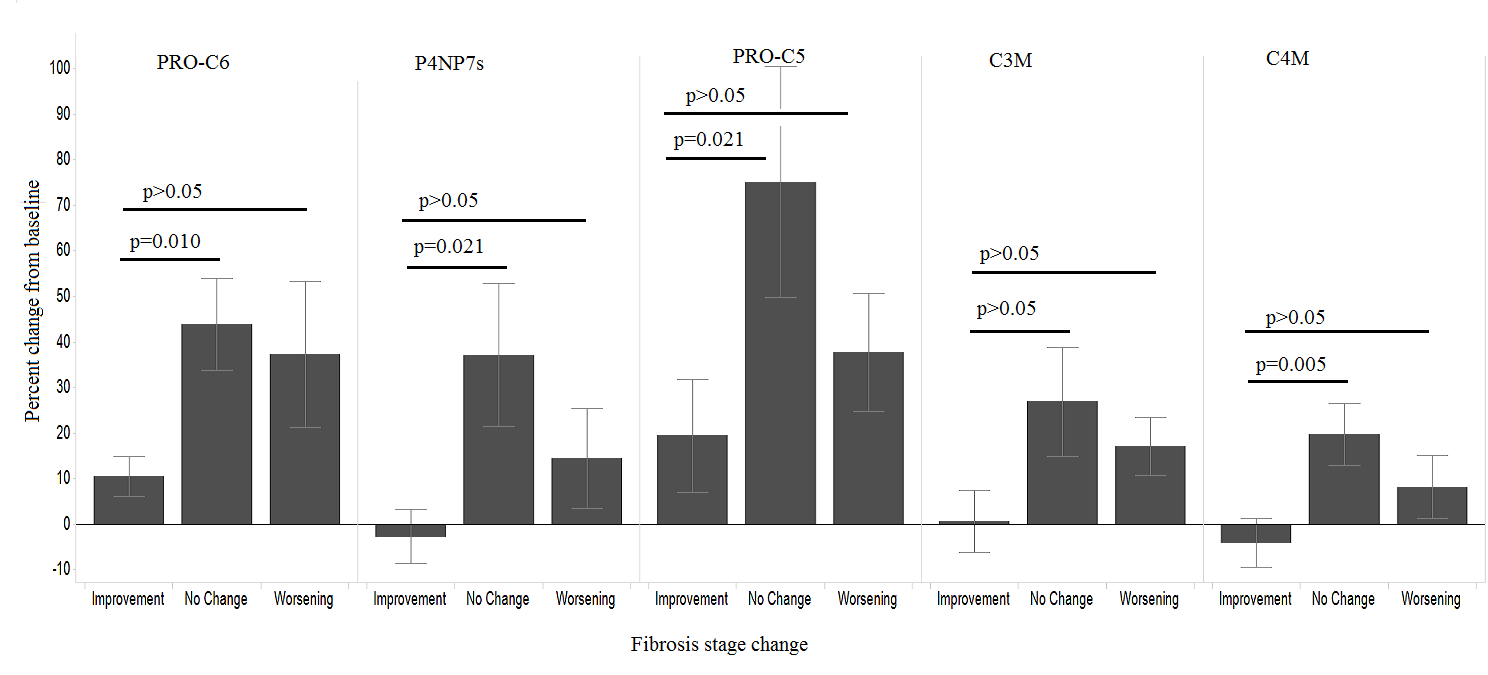

Supplement: Supplementary file 1 — Supplementary Material [file 41598_2018_30457_MOESM1_ESM.docx]
